# Modification of an RBF ANN-Based Temperature Compensation Model of Interferometric Fiber Optical Gyroscopes

**DOI:** 10.3390/s150511189

**Published:** 2015-05-13

**Authors:** Jianhua Cheng, Bing Qi, Daidai Chen, René Jr. Landry

**Affiliations:** 1 Marine Navigation Research Institute, College of Automation, Harbin Engineering University, Harbin 150001, China; E-Mails: ins_cheng@163.com (J.C.); chen.daidai@aalto.fi (D.C.); 2 LASSENA Laboratory, École de Technologie Superieure, 1100 Notre-Dame Street West, Montreal, QC H3C 1K3, Canada; E-Mail: ReneJr.Landry@etsmtl.ca; 3 Department of Biomedical Engineering and Computational Science, School of Science, Aalto University, Espoo 02150, Finland

**Keywords:** IFOG, temperature compensation, temperature product term, RBF ANN, MSD

## Abstract

This paper presents modification of Radial Basis Function Artificial Neural Network (RBF ANN)-based temperature compensation models for Interferometric Fiber Optical Gyroscopes (IFOGs). Based on the mathematical expression of IFOG output, three temperature relevant terms are extracted, which include: (1) temperature of fiber loops; (2) temperature variation of fiber loops; (3) temperature product term of fiber loops. Then, the input-modified RBF ANN-based temperature compensation scheme is established, in which temperature relevant terms are transferred to train the RBF ANN. Experimental temperature tests are conducted and sufficient data are collected and post-processed to form the novel RBF ANN. Finally, we apply the modified RBF ANN based on temperature compensation model in two IFOGs with temperature compensation capabilities. The experimental results show the proposed temperature compensation model could efficiently reduce the influence of environment temperature on the output of IFOG, and exhibit a better temperature compensation performance than conventional scheme without proposed improvements.

## 1. Introduction

With the rapid innovative development of science and technology, more and more Strap-down Inertial Navigation Systems (SINS) have been applied in the industrial production and the military field, such as navigation devices on robots, airplanes, ships and automobiles [1]. As the core sensor of SINS, Interferometric Fiber Optical Gyroscopes (IFOGs) play a critical role. IFOGs are used to measure the rotation information and to indicate the precise orientation of inertial measurement unit. As a result, the accuracy of an IFOG directly influences the navigation performance of the SINS [2].

IFOGs, which are manufactured based on semiconductor technology, are extremely sensitive to the ambient temperature [3]. For example, in the shipborne application, SINS are commonly installed in cabins which are located in central positions at the bottom of ships. Owing to suffering from the heat effect generated by the operation of other devices and external extreme climate, the temperature in these navigation cabins could vary from −10 °C to 40 °C, even with air conditioning [4]. As for IFOGs, the temperature fluctuation will definitely influence the physical characteristics of fiber loops, which drives the nonreciprocal phase error or the Temperature Shift Errors (TSE) in IFOGs [5,6]. For example, when the ambient temperature fluctuates by 10 °C, IFOGs with an output accuracy of ±0.01°/h will output a TSE of ±0.15°/h [7]. TSE may lead to the navigation error which accumulates at a much faster rate. In order to maintain the long-term operation accuracy of IFOG-based SINS in any scenario, it is essential and necessary to implement the temperature compensation within the IFOG.

The two most significant factors of IFOG temperature compensation models are the real-time property and universality [8,9]. Provided that temperature compensation models for IFOGs are non-linear models and prior models, Artificial Neural Network (ANN) is a well-suited choice to solve the above mentioned problems. Feng *et al.* [10] built a temperature compensation model based on Back-Propagation Artificial Neural Network (BP ANN). Temperature and temperature variation are selected as the inputs of their temperature compensation model and the IFOG outputs as the outputs of the temperature compensation model. This model has acceptable real-time properties, but there are some local minima in the BP ANN which make for a bad universality in the whole temperature range (−10 °C to 40 °C), even increasing the compensation error. With this objective to reduce the compensation error of BP ANN, Zhao *et al.* [11] improved the temperature compensation models using chaotic particle swarm optimization theory. However, the local minima problem in BP ANN is not solved efficiently, and the model is complicated enough to restrict its ability for real-time processing.

In order to overcome the local minima problem in BP ANN, a Radial Basis Function Artificial Neural Network (RBF ANN) is adopted. Shi *et al.* [12] built a temperature compensation model with RBF ANN with the operation-time of the IFOG as the input and the output errors of the IFOG as its output. The local minima problem can be avoided in this model. However, this scheme can only be used in some specific IFOGs and its compensation accuracy and repetitiveness are also limited. Aiming at increasing the compensation accuracy and increasing the repetitiveness, Yang *et al.* [13] used the temperature of fiber loops as the input of the model and IFOG outputs as its output to train a temperature compensation model based on RBF ANN. Thus, the local minima can be avoided, and the real output accuracy of the temperature compensation model is evidently improved by one order of magnitude, but due to the that there are not enough inputs, the RBF ANN structure has not been optimized very well and it cannot describe the nonlinear model approximately, which causes some uncertain and inestimable fluctuations, so Shen *et al.* [14] and Jin *et al.* [15] optimized the temperature compensation model based on RBF ANN with more inputs. They both have considered the temperature of fiber loops and temperature variation of fiber loops as inputs and chose some specific data as the centers of radial basis functions in the hidden layer with Ordinary Least Square (OLS), which ensures that the models have better compensation accuracy and better universality. Their model not only promotes the compensation accuracy by more than one order of magnitude, but also reduces the start-time by 50%. However, temperature variation during the training process is too small to ensure the compensation accuracy during the whole temperature range, which also causes that RBF ANN to not possess sufficient inputs to optimize the structure. Basically, sufficient kinds of inputs can optimize and improve the structure of the RBF ANN to approximate the nonlinear models more accurately. In summary, there is a trend to make more detailed classification of the temperature related terms to make RBF ANN compatible with more types of inputs to create more exact temperature compensation models.

This article is organized as follows: in Section 2, three temperature relevant factors of IFOG are extracted. Section 3 demonstrates the entire scheme for the temperature compensation models of IFOG based on input-modified RBF ANN. Section 4 shows the collected data used to train the RBF ANN and the test results of the IFOG-compensated temperature experiments. Section 5 evaluates the temperature compensation performances of the proposed scheme compared to previous traditional schemes. Section 6 presents the conclusions and benefits of the new method.

## 2. Modification of Temperature Compensation Models for IFOG

### 2.1. Traditional Temperature Compensation Models

**Figure 1:**
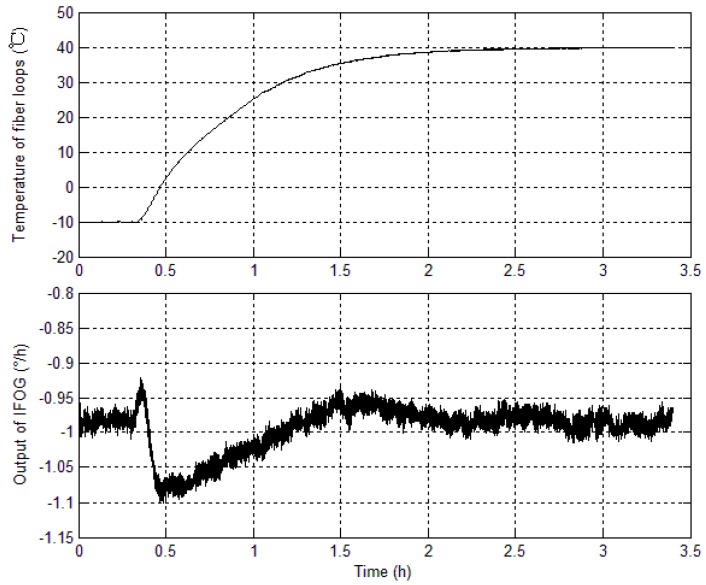
Curves for temperature of fiber loops and IFOG output.

As IFOGs are influenced by diverse internal and external sources of error, the navigation errors in the SINS solution are also diverse. To deduce a precise relationship between TSE and the temperature of fiber loops, temperature experiments are conducted to study the output properties of IFOGs. Assuming that in most applications IFOGs work in a temperature range from −10 °C to 40 °C, Figure 1 shows the curves for the relation between the temperature of fiber loops and IFOG outputs.

From Figure 1, the IFOG output varies with the fiber loop temperature. When the fiber loop temperature rises gradually, the IFOG output increases; when the fiber loop temperature stays stable, the IFOG output fluctuates around a certain value. This experiment clearly shows that the IFOG outputs are influenced by temperature. The next part of this section will examine the link between temperature and main IFOG characteristics.

**Figure 2:**
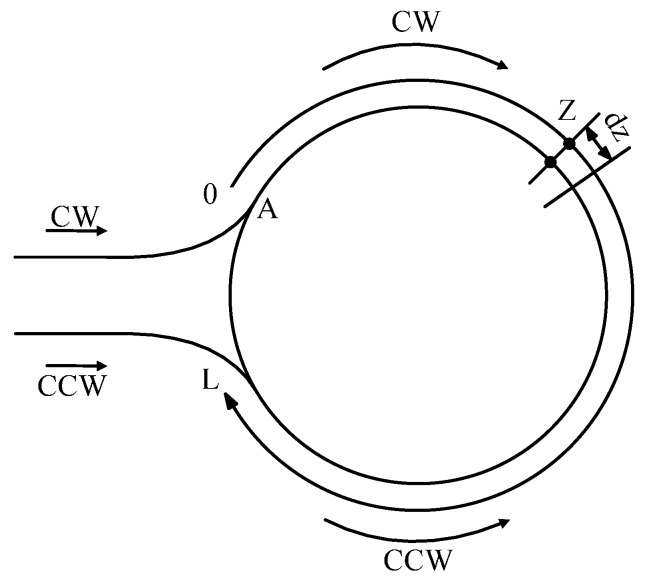
The schematic diagram of fiber loops.

According to the basic principle of IFOGs, assuming that two beams of interferometric light pass through a fiber loop whose total length is *L* (m) and the refractive index is *n*, one beam of light passes clockwise (CW) and the other one passes counter-clockwise (CCW). One end of the fiber loop is named A-end. They interfere each other at location *Z* that is *Z*(*m*) away from A-end [16,17,18]. The schematic diagram of fiber loops are shown in Figure 2.

The temperature at location *Z* is studied in temperature experiments. When the temperature at location *Z* is varying, the thermal induced nonreciprocity phase delay caused by temperature variation can be described by [19]:

(1) $$\Delta \phi_{i}=\frac{2\text{π}n}{\lambda c}(\frac{\partial n}{\partial t}+n{\text{α}}_{T})\frac{\partial T}{\partial t}(L-2z)dz$$

where, ∂*T*(*z*, *t*)/∂*t* is the temperature variation in the place *Z*. *n* is the refractive index of fiber. λ is the light wavelength. α*_T_* is the thermal expansion coefficient of fibers. *C* is the light speed in y waveguide. According to Equation (1), ∆ϕ*_i_* is related to the temperature of the fiber loops and the temperature variation of the fiber loops. Hence, traditional temperature compensation models are built based on both of them, and the temperature compensation models for IFOG are as follows:

(2) $$\Delta \phi_{i}=f(T,\Delta T)$$

### 2.2. Modified Temperature Compensation Models

As it is known, the thermal induced nonreciprocity phase delay ∆ϕ*_i_* is inherent to all kinds of fiber loops. From Equation (1), the thermal induced nonreciprocity phase delay ∆ϕ*_i_* is related to the temperature of fiber loops *T*, temperature variation of fiber loops ∂*T*/∂*t* and refractive index variation of fiber loops ∂*n*/∂*t* [20].

Refractive index variation of fiber loops ∂*n*/∂*t* is commonly considered as constant all over the IFOG, however, it is usually affected by ambient temperature and the strain condition of fibers. The refractive index of fiber loops is denoted by *n*(λ, *T*, ε), where λ is the light wavelength, and *T* is the environmental temperature, and ε is the stress on fiber loops. Assuming that there is no specific stress on the fiber loop, ε can be ignored. When the temperature remains at *T*_0_ stably and a beam of light passes through the fiber loops, the refractive index of the fiber loops is *n*(λ, *T*_0_). According to Taylor’s formula, the refractive index of the fiber loops *n*(λ, *T*_0_) can be expended as:

(3) $$n(\lambda ,T)=n(\lambda ,T_{0})+\frac{\partial n}{\partial T}(T-T_{0})$$

The temperature coefficient of refractive index of the fiber loops can be expressed as:

(4) $$C_{T}=\frac{1}{n(\lambda ,T_{0})}\frac{\partial n}{\partial T}$$

To show how many temperature relevant terms influence the fiber loops, a comparative optical path experiment will be done. A fiber loop whose length is *L* (m) will be tested, and several optical paths will be compared at different ambient temperatures. When the ambient temperature is *T*_0_, the optical path equation is given by:

(5) $$L(T_{0})n(\lambda ,T_{0})=L_{0}(T_{0})n(\lambda ,T_{0})+x$$

where, *L*_0_(*T*_0_) is the measured total length of optical path, and *L*_0_(*T*_0_) is the real total length of optical path, and *x* is the distance from one end of the fiber loop to the scanning mirror. According to the thermal expansion effect, when the ambient temperature rises, the length of optical path will extend. A new optical path equation can be written as:

(6) $$L(T_{0})[1+{\text{α}}_{T}(T-T_{0})]n(\lambda ,T_{0})[1+C_{T}(T-T_{0})]=L_{0}(T_{0})n(\lambda ,T_{0})+x+\Delta x$$

where, α*_T_* = 5.5 × 10^−7^/°C is the thermal expansion coefficient of fibers, and ∆*x* is the micro-variation of the distance from the end of the fiber loop to the scanning mirror. From Equations (4)–(6), ∆*x* can be obtained as follows:

(7) $$\Delta x=L(T_{0})n({\text{α}}_{T}+C_{T})(T-T_{0})$$

From Equation (7), the temperature coefficient of refractive index of fibers *C_T_* can be expressed as:

(8) $$C_{T}=\frac{\Delta x}{L(T_{0})n(T-T_{0})}-{\text{α}}_{T}$$

From Equations (4) and (8), we have:

(9) $$\frac{\Delta x}{L(T_{0})n(T-T_{0})}-{\text{α}}_{T}=\frac{1}{n}\frac{\partial n}{\partial T}$$

Hence, Equation (9) can be simplified as follows:

(10) $$\frac{\partial n}{\partial T}=\frac{\Delta x}{L(T_{0})(T-T_{0})}-n{\text{α}}_{T}$$

Then, from Equations (1) and (10), we obtain:

(11) $$\Delta \phi_{i}=\frac{2\text{π}n\Delta T}{\lambda c}(L-2z)dz$$

where, ∆*T* = ∂*T*/∂*t* is the temperature variation. The thermal expansion equation is described by:

(12) $$dz=L-L_{0}={\text{α}}_{T}L_{0}T$$

From Equations (11) and (12), a new simultaneous equation is obtained:

(13) $$\Delta \phi_{i}=\frac{2\text{π}n{\text{α}}_{T}L_{0}[({\text{α}}_{T}T+1)L_{0}-2z]}{\lambda c}(T\times \Delta T)$$

**Figure 3:**
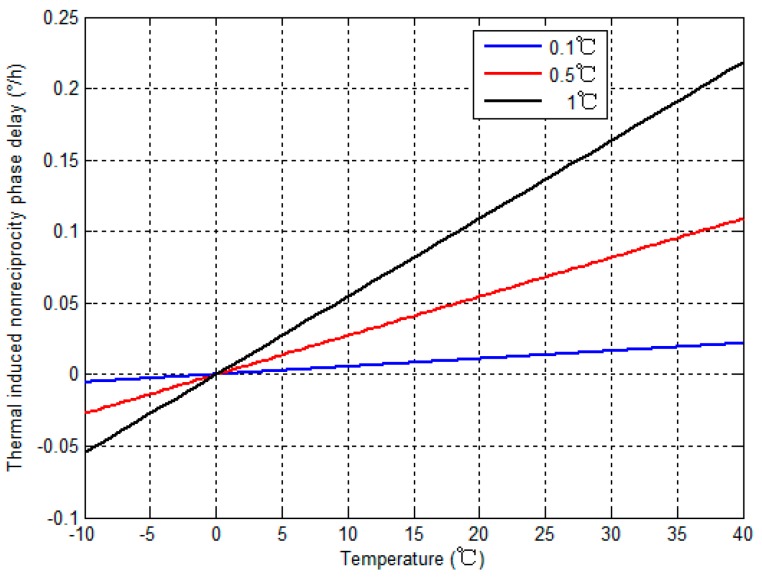
Thermally induced nonreciprocity phase delay in simulation experiments.

From Equation (13), ∆ϕ*_i_* is related to the fiber loop temperature *T*, temperature variation of the fiber loop ∆*T* and temperature product term of the fiber loop *T* × ∆*T*. To identify the influence that *T*, ∆*T* and *T* × ∆*T* have on ∆ϕ*_i_*, respectively, three simulation experiments are conducted with the conditions that the length of the fiber loop is 1000 m, and the thermal expansion coefficient of the fibers is 5.5 × 10^−7^/°C, and light whose wavelength is 1550 nm passes through the fiber loop. The summary of the experimental conditions are as follows: when the ambient temperature varies in intervals which are shown in Table 1 from −10 °C to 40 °C, the thermal induced nonreciprocity phase delay ∆ϕ*_i_* in a place which is 0.25*L*_0_ away from A-end can be shown in Figure 3.

**Table 1:** Simulation experimental conditions.

Experimental Conditions	1st	2nd	3rd
Temperature range	−10 °C to 40 °C	−10 °C to 40 °C	−10 °C to 40 °C
Temperature varied interval	0.1 °C	0.5 °C	1 °C

From Figure 3, the thermal induced nonreciprocity phase delay ∆ϕ*_i_* related with *T*, ∆*T* and *T* × ∆*T* affects the IFOG output remarkably seriously, and the maximal value of the thermally induced nonreciprocity phase delay ∆ϕ*_i_* is more than 0.2 °/h. Moreover, ∆ϕ*_i_* will rise as *T*, ∆*T* and *T* × ∆*T* increase. In conclusion, temperature relevant terms such as *T*, ∆*T* and *T* × ∆*T* are critical and non-negligible factors to design temperature compensation models for IFOGs. Hence, the modified temperature compensation model for IFOG can be expressed more accurately as follows:

(14) $$\Delta \phi_{i}=f(T,\Delta T,T\times \Delta T)$$

## 3. Design of Modified Temperature Compensation Models Based on RBF ANN

It is a prior process to establish temperature compensation models for IFOG which are nonlinear. According to the fundamental theories concerning artificial neural networks, an RBF ANN is suitable to build prior models for temperature compensation [21].

**Figure 4:**
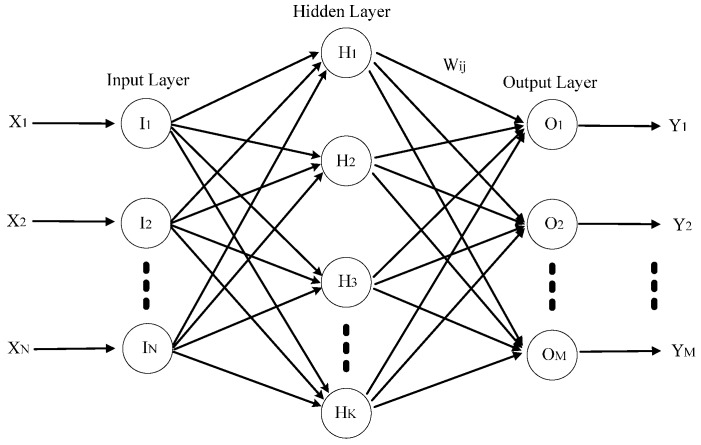
The structure of an RBF ANN.

RBF ANN is a kind of 3-layer neural network, which is composed of an input layer, a hidden layer and an output layer. The neurons (*I_i_*, *H_i_* and *O_i_*) can be divided to several groups. These neurons groups are renamed as nervous layers, and they are considered as basic computational units. The input signals are transmitted between neurons and nervous layers. Figure 4 shows the structure of an RBF ANN.

Where, *X_i_*(*i* = 1, …, *N*) is the *i*th input of RBF ANN, and *Y_i_*(*i* = 1, …, *M*) is the *i*th output of RBF ANN, and *Ii*(*i* = 1, …, *N*) is the ith neuron of the input layer, and *H_i_*(*i* = 1, …, *K*) is the *i*th neuron of the hidden layer, and *O_i_*(*i* = 1, …, *M*) is the *i*th neuron of the output layer. Inputs are divided into several groups by kernel functions. Usually, a Gaussian function is chosen as the kernel function for neurons in the hidden layer. The Gaussian function is described by:

(15) $$\phi_{j}(x)=e^{-{\left\|x-c_{j}\right\|}^{2}/2\sigma_{j}^{2}}\;j=(1,2,\cdots ,K)$$

The most important point for an RBF ANN is how to fix the center *c_j_* and the width σ*_j_* of kernel function. Figure 4 shows N neurons in the input layer, K neurons in the hidden layer and M neurons in the output layer. From Equation (15), ϕ*_j_*(*x*) is the output of the *j*th neuron in the hidden layer, and *c_j_* is the center vector of kernel function of the *j*th neuron in the hidden layer, and *x* is a *N*-dimensional input vector, and σ*_j_* is the width of Gauss function of the *j*th neuron in the hidden layer, and ||x − *c_j_*|| is the distance between input vector and the center vector of Gauss function. Then, output of the RBF ANN can be expressed as:

(16) $$y_{j}=\sum_{j=1}^{K}W_{ij}\phi_{j}(x),\;i=1,2,\cdots ,M$$

where, *y_j_* is the output of the *j*th neuron in the output layer, *W_ij_* is the weight between the *i*th neuron in the output layer and the *j*th neuron in the hidden layer. The sample set can be divided to several groups by RBF ANN with Equation (15). Meanwhile, the difference between the actual output and the desirable output decides if *c_j_* and σ*_j_* will be adjusted. The adjusted magnitudes of *W_ij_*, *c_j_* and σ*_j_* are ∆*W_ij_*, ∆*c_j_* and ∆σ*_j_*, respectively. Their expressions are given by:

(17) $$\Delta W_{ij}=\eta_{w}(d_{i}-y_{i})\phi_{j}(x)$$

(18) $$\Delta c_{j}=\eta_{c}\sum_{i=1}^{M}\left[(d_{i}-y_{i})W_{ij}\right]\frac{\left(x-c_{j}\right)}{\sigma_{j}^{2}}\phi_{j}(x)$$

(19) $$\Delta \sigma_{j}=\eta_{\sigma }\sum_{i=1}^{M}\left[(d_{i}-y_{i})W_{ij}\right]\frac{{\left\|x-c_{j}\right\|}^{2}}{\sigma_{j}^{3}}\phi_{j}(x)$$

When an RBF ANN is being trained, *W_ij_*, *c_j_* and σ*_j_* will be adjusted separately with previous Equations (17)–(19). After being adjusted several times, the RBF ANN outputs will meet the design requirement.

The RBF ANN has the two following advantages:

(1) The RBF ANN can avoid local minima. Owing to the fact that an RBF ANN works on the basis of a Gaussian function, the current results are optimal in the whole range, even under complex conditions that the error gradient approximate to zero in some flat areas.
(2) According to the Kolmogorov theorem, any feedforward neural network which consists of three nervous layers can approximate any continuous function in desired accuracy. Considering the universality and real-time implementation requirements, the structure of temperature compensation models should be as simple as possible. Hence, an RBF ANN does well in increasing real-time and universality of temperature compensation models for IFOGs [22].

Hence, according to Equation (2), the traditional temperature compensation models for IFOG based on an RBF ANN can be written as follows:

(20) $$\Delta \phi_{i}=ANN_{RBF}(T,\Delta T)$$

However, it is deficient that only *T* and ∆*T* are added up to temperature compensation models. More relevant inputs will complete and improve the structure of RBF ANN. With more relevant inputs, the center *c_j_* of kernel function and the width σ*_j_* of kernel function can be configured more precisely, and the weights between the hidden layers and the output layers can be adjusted more accurately. Meanwhile, from Equation (14), it was shown that the temperature of fiber loops *T*, temperature variation of fiber loops ∆*T* and temperature product term of fiber loops *T* × ∆*T* are essential and necessary to establish robust and precise temperature compensation models. Hence, the modified temperature compensation models can be expressed as:

(21) $$\Delta \phi_{i}=ANN_{RBF}(T,\Delta T,T\times \Delta T)$$

## 4. Experimental Verification of Modification for Temperature Compensation Model

In order to test the modification for temperature compensation models of an IFOG-based RBF ANN, temperature experiments which can test all different kinds of IFOG in diverse situations should be designed and performed. Considering that fiber loops are temperature-sensing devices and IFOGs rely on their performance the stability and performance of IFOGs are strongly related to the temperature relevant terms of fiber loops. With this regard, the main purpose of temperature experiments is to detect the temperature relevant terms of fiber loops. Compared to the traditional temperature compensation models, there are several implementation challenges in modification for the temperature compensation model. They are shown as follows:

(1) According to Equation (21), the modified temperature compensation model based on RBF ANN needs a bigger training set to increase its precision, which requires that the temperature experiments supply a bigger training set, and reducing the rate of temperature variation is a useful way of supplying more training data. The air gap clearance between temperature environmental chamber (TEC) and IFOG could slow down the rate of heat transfer. Hence, it is necessary that a bigger temperature environmental chamber be used to slow down the rate of temperature variation.
(2) According to plenty of random experimental temperature and IFOG data, when the variation of the ambient temperature is less than 0.1 °C, the IFOG outputs remain almost stable. However, when the variation of ambient temperature is more than 0.1 °C, the IFOG outputs will change. Aiming at ensuring compensation accuracy, the temperature measurement is more than one order of magnitude exact than the ambient temperature.
(3) According to Equations (2) and (21), the difference between the traditional temperature compensation model and the modified temperature compensation model is *T* × ∆*T* which is calculated with *T* and ∆*T*, and the calculation never costs extra memory or other resources. The most important point is that the real-time will never be reduced at all. Meanwhile, it is easy to realize *T* × ∆*T* in software directly.

However, all of implementation challenges can be overcame by choosing suitable devices and algorithms. Still, they will not influence the real-time and universality of the modified temperature compensation model, and it is easy to realize modifications for a temperature compensation model.

**Figure 5:**
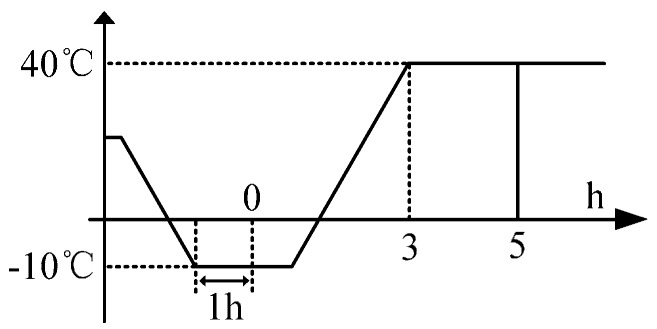
Experimental sequence.

Above all, a TEC with a high-precision turning table whose performances are a temperature measuring accuracy of 0.01 °C and temperature control accuracy of 0.1 °C should be used to test three random free-running IFOGs, and each IFOG will be tested five times. Figure 5 shows the experimental sequence [23]. The experimental procedures are as follows:

(1) The ambient temperature goes down to 10 °C and must stay stable for 1 h, and then the temperature of fiber loops and IFOG outputs will be recorded;
(2) The ambient temperature goes up progressively to 40 °C and the temperature of fiber loops and IFOG outputs will be kept stable for 2 h and then be recorded;
(3) Redo the same experiment five times, and calculate the average fiber loop temperature and IFOG output in order to reduce the accidental probability error;

All three IFOGs are installed on the high-precision turning table in the TEC under static base conditions [24,25,26]. Before starting the experiments the turning table should be set to a known direction *A*, and the IFOG cannot be moved until the experiments are finished.

**Figure 6:**
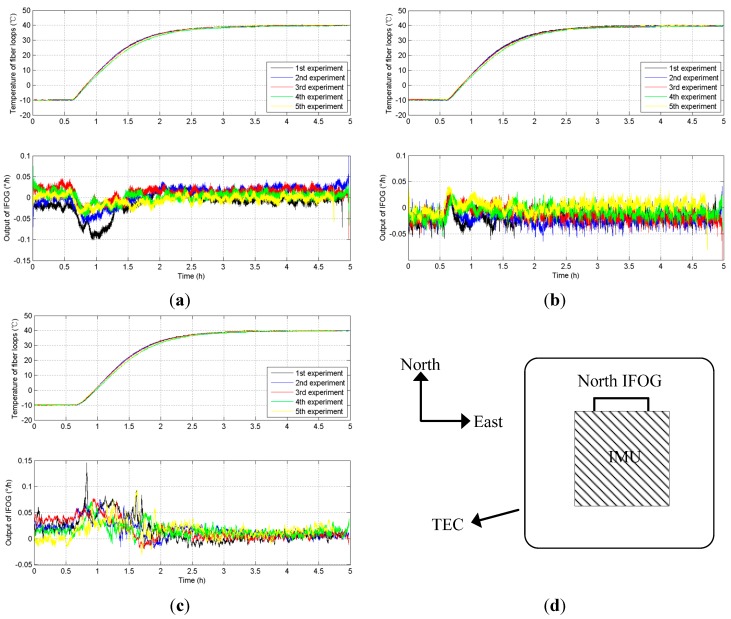
Five groups of fiber loop temperatures and IFOG outputs. (**a**) Test results from NO.1 IFOG; (**b**) Test results from NO.2 IFOG; (**c**) Test results from NO.3 IFOG; (**d**) The placement of the tested IFOG.

We select the FOG and orient its sensitive axis along with the true north, as shown in Figure 6d. The fiber loop temperatures and IFOG outputs based on five diverse temperature experiments from NO.1 IFOG, NO.2 IFOG, and NO.3 IFOG, respectively, are shown in Figure 6a–c. From Figure 6, when the temperature rises, the magnitudes of the IFOG outputs increase; when the temperature remains stable, the magnitudes of the IFOG outputs fluctuate around a certain constant. Output trends from NO.1 IFOG, NO.2 IFOG, and NO.3 IFOG are almost similar, and they are closely related to the temperature of the fiber loops, and the IFOG outputs vary with the temperature variations of fiber loops.

According to Equation (14), the thermally-induced nonreciprocity phase delay ∆ϕ*_i_* is related to the fiber loop temperature *T*, fiber loop temperature variation ∆*T* and temperature product term *T* × ∆*T*. The temperature variation of the fiber loops ∆*T* can be expressed by Equation (22):

(22) $$\Delta T=\{\begin{array}{c} \begin{array}{ll} 0 & \begin{array}{cc} \begin{array}{cc} & i=1 \end{array} & \end{array} \end{array}\\ \begin{array}{cc} T_{i}-T_{i-1} & i=2\cdots N \end{array} \end{array}$$

**Figure 7:**
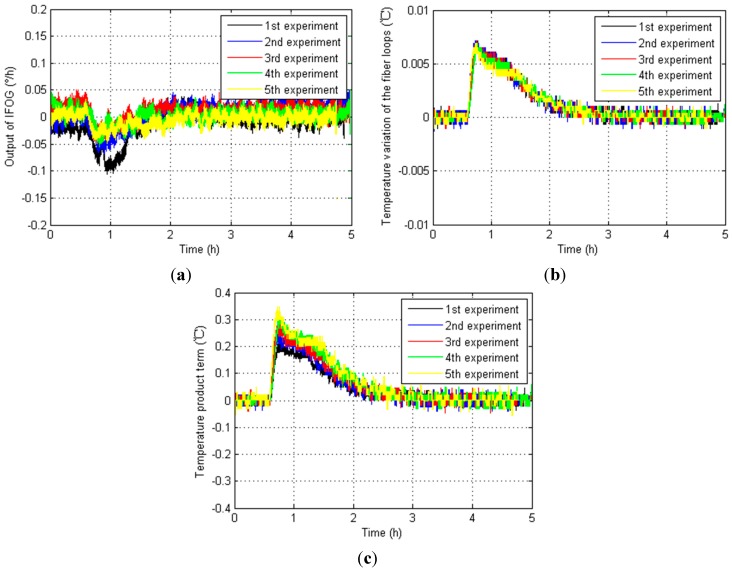
Relevant test results from NO.1 IFOG. (**a**) IFOG outputs; (**b**) Fiber loop temperature variation ∆*T*; (**c**) Temperature product term *T* × ∆*T*.

**Figure 8:**
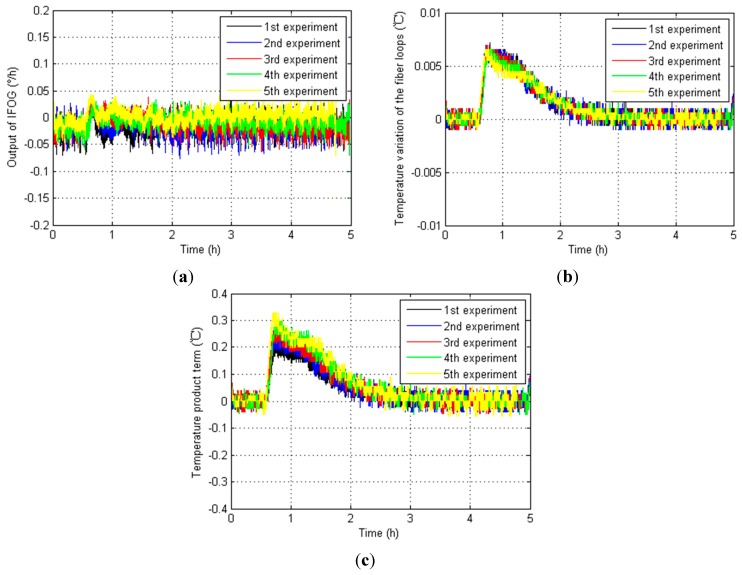
Relevant test results from NO.2 IFOG. (**a**) IFOG outputs; (**b**) Fiber loop temperature variation ∆*T*; (**c**) Temperature product term *T* × ∆*T*.

**Figure 9:**
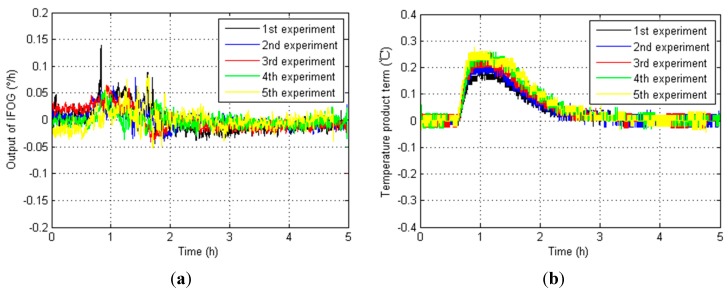

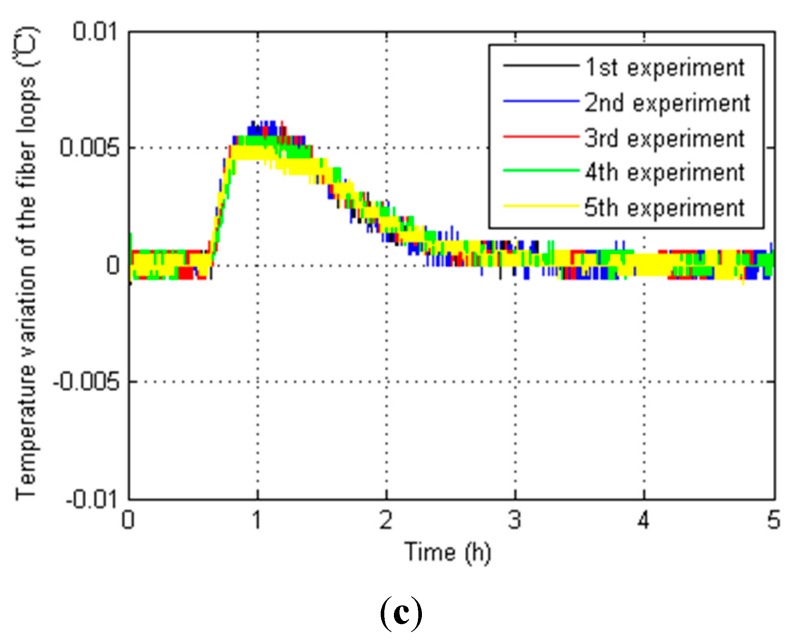
Relevant test results from NO.3 IFOG. (**a**) IFOG outputs; (**b**) Fiber loop temperature variation ∆*T*; (**c**) Temperature product term *T* × ∆*T*.

Furthermore, the IFOG outputs, fiber loop temperature variation ∆*T* and temperature product term *T* × ∆*T* in five temperature experiments from NO.1 IFOG, NO.2 IFOG and NO.3 IFOG are shown in Figure 7, Figure 8 and Figure 9, respectively.

According to Figure 7, Figure 8 and Figure 9, the magnitudes of the IFOG outputs are proportional to the temperature variation of the fiber loops ∆*T*, and they fall gradually as the temperature variation of the fiber loops ∆*T* decreases to zero. When it decreases to zero, the IFOG outputs fluctuate around some certain constant values. Similarly, the temperature product term *T* × ∆*T* has a homologous performances. That means the fiber loop temperature variation ∆*T* and temperature product term *T* × ∆*T* influence the performance of IFOGs, and both of them have to be considered as necessary factors for any IFOG temperature compensation model, so the modified temperature compensation models are described by:

(23) $$\Delta E=ANN_{RBF}(T,\Delta T,T\times \Delta T)$$

Hence, according to Equation (23), the temperature compensation model based on RBF ANN should be trained step by step as follows:

(1) Conduct two different groups of diverse temperature experiments, and one group is chosen randomly as a training set, and the other one is chosen as a verification set;
(2) An error set *Q* will be obtained from the subtraction between the average of the training set and the training set;
(3) Use *T*, ∆*T*, *T* × ∆*T* and *Q* to train the RBF ANN until the errors between the outputs of the trained RBF ANN and the error set *Q* meet the requirements;
(4) The compensated results will be obtained from subtraction between the RBF ANN outputs and the corresponding temperature compensation model outputs.

**Figure 10:**
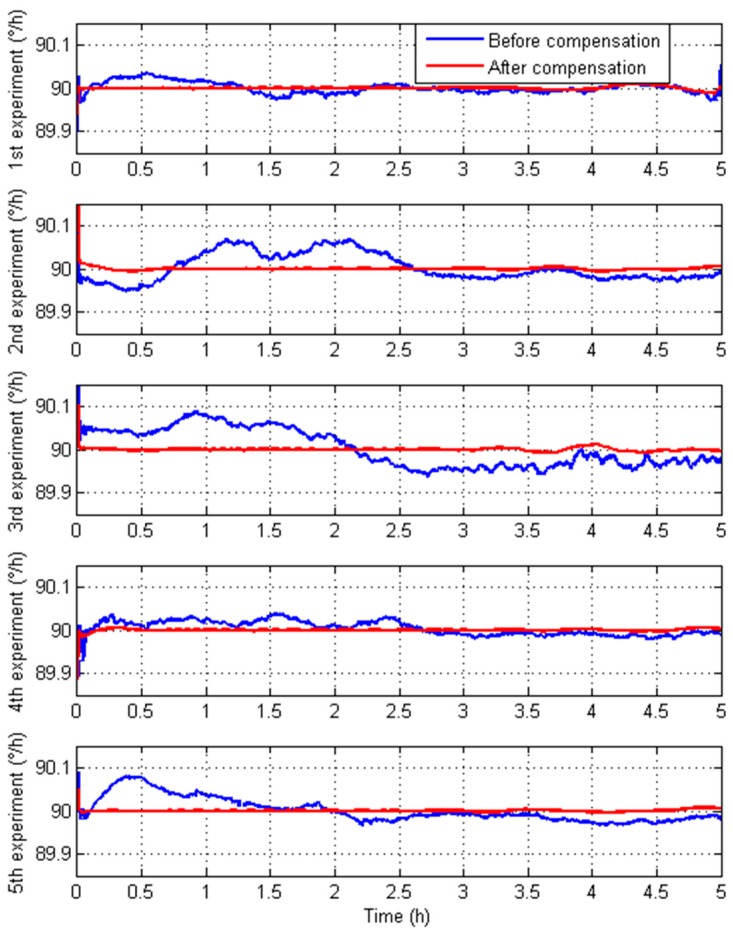
The primary outputs of IFOG and the compensated outputs of IFOG.

To illustrate the validity of the temperature compensation models which are built according to the training steps, one of three IFOGs mentioned will be chosen randomly and tested in temperature experiments, and temperature compensation models will be trained with the test results from these temperature experiments. With one of the objectives being to ensure the universality of the method, the IFOGs are precisely installed on a high-precision turning table in TEC, and it will be rotated to the east where *A* = 90°, and be tested in another five temperature experiments [27,28,29]. Figure 10 shows the primary IFOG outputs and the compensated IFOG outputs.

From Figure 10, the temperature compensation model could efficiently reduce the temperature shift errors. The IFOG temperature compensation model is estimated by the Mean Square Deviation (MSD) estimation formula. The MSD estimation formula is given by:

(24) MSD=MSE(d−A)

where MSD is the Mean Square Deviation, and MSE is the mean square error algorithm, and *d* is the desired sample, and *A* is the known rotation angle of the high-precision turning table. This estimation formula uses the errors between the desired sample and the true value of the desired sample to indicate their relativity. The smaller MSD is, the more closely they are related to each other. MSD values before and after compensation are shown in Table 2.

**Table 2:** MSD before and after compensation.

NO.	MSD before Compensation	MSD after Compensation
1st experiment	1.72 × 10^−4^	1.24 × 10^−5^
2nd experiment	9.90 × 10^−4^	1.76 × 10^−5^
3rd experiment	1.80 × 10^−3^	2.05 × 10^−5^
4th experiment	2.67 × 10^−4^	1.33 × 10^−5^
5th experiment	9.29 × 10^−4^	0.78 × 10^−5^

Table 2 shows that the mean square deviation has obviously become much smaller. In conclusion, the modified temperature compensation model can reduce TSE exactly and effectively.

## 5. Comparison of Experimental Results Before and After Modification

Aiming at demonstrating that the temperature compensation model after modification performs better than that before modification, the experimental results before and after modification will be compared, and the temperature compensation models are shown as follows:

(25) $$\Delta E_{BM}=ANN_{RBF}(T,\Delta T)$$

(26) $$\Delta E_{AM}=ANN_{RBF}(T,\Delta T,T\times \Delta T)$$

**Figure 11:**
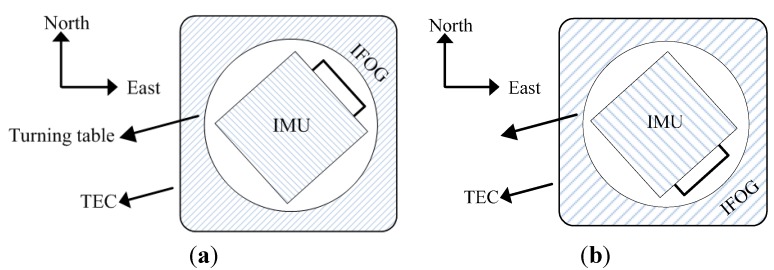
The placement of the tested IFOG. (**a**) The placement of NO.1 IFOG with a rotation angle *A*_1_ = 45°; (**b**) The placement of NO.2 IFOG with a rotation angle *A*_2_ = 135°.

**Figure 12:**
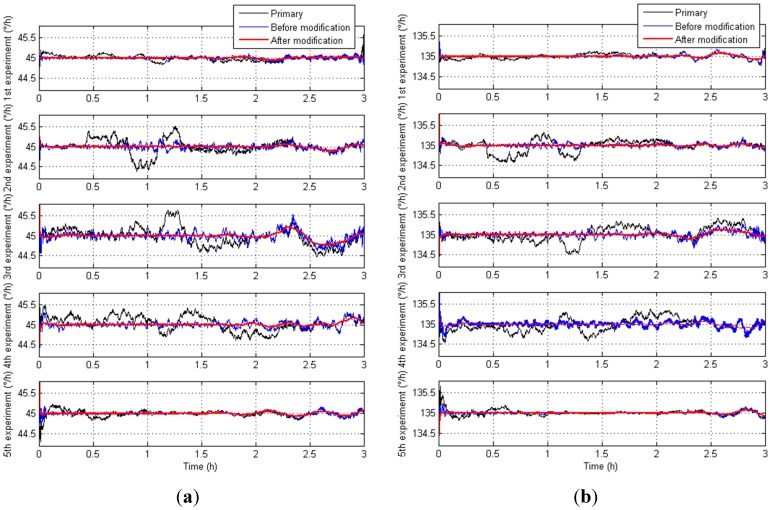
The primary outputs and the compensated outputs from diverse IFOGs. (**a**) The primary outputs and the compensated outputs from NO.1 IFOG; (**b**) The primary outputs and the compensated outputs from NO.2 IFOG.

To verify the universality of the temperature compensation model, two other IFOGs are chosen and tested in five diverse temperature experiments [30,31,32]. Both of them are installed in TEC on a high-precision turning table, and they are separately placed randomly with a rotation angle *A*_1_ = 45° and with a rotation angle *A*_2_ = 135° to ensure the universality of the temperature experiments. The representation of the tested IFOG orientations are shown in Figure 11a,b. The primary IFOG outputs, the compensated outputs based on Equation (25) and the compensated outputs based on Equation (26) are shown in Figure 12a,b, respectively.

From Equation (24), the MSD estimation formula can be transformed to estimate the results. They are described by:

(27) $$MSD_{1}=MSE(d-A_{1})$$

(28) $$MSD_{2}=MSE(d(T,\Delta T)-A_{1})$$

(29) $$MSD_{3}=MSE(d(T,\Delta T,T\times \Delta T)-A_{1})$$

(30) $$MSD_{4}=MSE(d-A_{2})$$

(31) $$MSD_{5}=MSE(d(T,\Delta T)-A_{2})$$

(32) $$MSD_{6}=MSE(d(T,\Delta T,T\times \Delta T)-A_{2})$$

Table 3 shows the MSD based on Equations (27)–(29) from NO.1 IFOG. Table 4 shows MSD based on Equations (30)–(32) from NO.2 IFOG.

**Table 3:** MSD from NO.1 IFOG.

NO.	*MSD*_1_	*MSD*_2_	*MSD*_3_
1st experiment	5.40 × 10^−3^	1.10 × 10^−3^	2.02 × 10^−4^
2nd experiment	3.37 × 10^−2^	4.60 × 10^−3^	9.93 × 10^−4^
3rd experiment	5.79 × 10^−2^	1.93 × 10^−2^	7.70 × 10^−3^
4th experiment	3.87 × 10^−2^	9.40 × 10^−3^	2.90 × 10^−3^
5th experiment	1.00 × 10^−2^	3.80 × 10^−3^	1.40 × 10^−3^

**Table 4:** MSD from NO.2 IFOG.

NO.	*MSD*_4_	*MSD*_5_	*MSD*_6_
1st experiment	5.40 × 10^−3^	2.10 × 10^−3^	6.05 × 10^−4^
2nd experiment	2.45 × 10^−2^	2.80 × 10^−3^	7.48 × 10^−4^
3rd experiment	3.51 × 10^−2^	8.20 × 10^−3^	3.30 × 10^−3^
4th experiment	3.11 × 10^−2^	8.20 × 10^−3^	2.70 × 10^−3^
5th experiment	8.40 × 10^−2^	4.20 × 10^−3^	1.90 × 10^−3^

From Figure 12, the modified temperature compensation model can deduce the temperature shift errors more precisely and more universally, and the compensated IFOG outputs are not mainly influenced by the temperature. From Table 3 and Table 4, the mean square deviation after modification is smaller than that before modification, and much smaller than the primary output. In a word, the real output accuracy of an IFOG after modification has been raised and improved better than that before modification with the proposed modified temperature compensation model.

## 6. Conclusions

Accuracy, real-time and universality are very important for temperature compensation models for IFOGs. In this paper, we put forward a modification of an RBF ANN based on temperature compensation models for IFOGs. The temperature compensation model after modification based on the temperature of fiber loops *T*, temperature variation of fiber loops ∆*T* and temperature product term *T* × ∆*T* can deduce temperature shift errors more exactly, and the compensation accuracy is increased by more than one order of magnitude.
